# Advancements in Ligand-Based Virtual Screening through the Synergistic Integration of Graph Neural Networks and Expert-Crafted Descriptors

**DOI:** 10.1021/acs.jcim.5c00822

**Published:** 2025-05-14

**Authors:** Yunchao Liu, Rocco Moretti, Yu Wang, Ha Dong, Bailu Yan, Bobby Bodenheimer, Tyler Derr, Jens Meiler

**Affiliations:** † Department of Computer Science, 5718 Vanderbilt University, 2201 West End Ave, Nashville, Tennessee 37235, United States; ‡ Department of Chemistry, Center for Structural Biology, Vanderbilt University, 2201 West End Ave, Nashville, Tennessee 37235, United States; § School of Computer and Data Sciences, 3265 University of Oregon, 1585 East 13th Avenue, Eugene, Oregon 97403, United States; ∥ Department of Neural Science, 1180 Amherst College, 220 South Pleasant Street, Amherst, Massachusetts 01002, United States; ⊥ Department of Biostatistics, Vanderbilt University, 2201 West End Ave, Nashville, Tennessee 37235, United States; # Department of Computer Science, Electrical Engineering and Computer Engineering, Vanderbilt University, 2201 West End Ave, Nashville, Tennessee 37235, United States; ∇ Department of Computer Science, Data Science Institute, Vanderbilt University, 2201 West End Ave, Nashville, Tennessee 37235, United States; ○ Institute of Drug Discovery, Leipzig University Medical School, Härtelstraße 16-18, Leipzig 04103, Germany; ◆ Center for Scalable Data Analytics and Artificial Intelligence (ScaDS.AI), Humboldtstraße 25, Leipzig 04105, Germany

## Abstract

The fusion of traditional chemical descriptors with graph neural networks (GNNs) offers a compelling strategy for enhancing ligand-based virtual screening methodologies. A comprehensive evaluation revealed that the benefits derived from this integrative strategy vary significantly among different GNNs. Specifically, while GCN and SchNet demonstrate pronounced improvements by incorporating descriptors, SphereNet exhibits only marginal enhancement. Intriguingly, despite SphereNet’s modest gain, all three models-GCN, SchNet, and SphereNet-achieve comparable performance levels when leveraging this combination strategy. This observation underscores a pivotal insight: sophisticated GNN architectures may be substituted with simpler counterparts without sacrificing efficacy, provided that they are augmented with descriptors. Furthermore, our analysis reveals a set of expert-crafted descriptors’ robustness in scaffold-split scenarios, frequently outperforming the combined GNN-descriptor models. Given the critical importance of scaffold splitting in accurately mimicking real-world drug discovery contexts, this finding accentuates an imperative for GNN researchers to innovate models that can adeptly navigate and predict within such frameworks. Our work not only validates the potential of integrating descriptors with GNNs in advancing ligand-based virtual screening but also illuminates pathways for future enhancements in model development and application. Our implementation can be found at https://github.com/meilerlab/gnn-descriptor.

## Graphical abstract

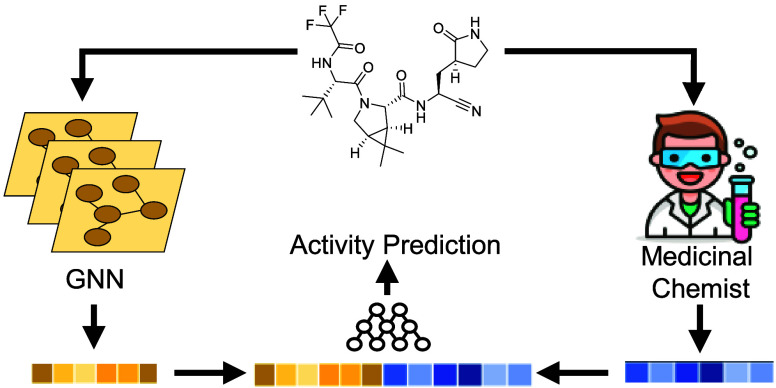

## 1 Introduction

Virtual screening is a major way to supplement traditional high-throughput screening (HTS) for cost- and time-efficient drug discovery.^1^ Two major branches of virtual screening exist: ligand-based and structure-based. For the application of structure-based methods, detailed knowledge of the target’s structure is essential, typically acquired through experimental methods such as X-ray crystallography or nuclear magnetic resonance (NMR). In cases where experimental data is lacking, computational predictions like homology modeling are employed to infer the three-dimensional configurations of targets. Recently, there are many AI-driven protein structure prediction tools available as well, such as AlphaFold,^2^ RosettaFold,^3,4^ ESMFold.^5^

This work focuses on the ligand-based method, for situations where the target structure remains unknown or cannot be computationally predicted. These methods depend on the knowledge of previously identified active compounds that bind to the target, leveraging this information to identify potential new drugs.^6^ Even in the age that computational protein structure prediction tools are available, ligand-based approaches are needed for several reasons. First, while structure prediction tools have made remarkable progress, there are still limitations in their ability to accurately predict all protein structures, especially for proteins with highly dynamic regions and transient conformations. The ligand-based method does not require structural information, making it valuable for targets where high-quality structures are not available. Second, ligand-based methods can sometimes be faster and less resource-intensive than structure-based methods, especially in the early stages of drug discovery. They allow researchers to quickly screen vast chemical spaces or compound libraries to identify potential hits without detailed structural information. Third, some targets have multiple or flexible binding sites that can be challenging to characterize with structure-based methods alone. Ligand-based methods can help identify ligands that interact with such targets by leveraging data from known active compounds without relying on a fixed three-dimensional (3D) structure.

Meanwhile, numerous studies applied graph neural networks (GNNs) to molecule-related tasks, given the intrinsic graph nature of molecules.^7–13^ While some of those tasks achieve good results, several factors still make GNN for molecule representation learning challenging. First, data available for training in drug discovery campaigns is usually limited due to the high cost of experimental assays. Second, GNNs typically have difficulty learning molecular-level features due to their limited receptive field or learning nonadditive molecular-level features such as total polar surface area. Third, GNN intrinsically suffers from problems such as oversmoothing^14^ and oversquashing^15^ that introduce information loss in obtaining the global learned embedding from the atomic features.

As a solution, integrating the expert knowledge in the GNN workflow has become a new trend.^16^ Expert knowledge can help supplement the data-hungry GNNs with prior knowledge to increase data efficiency and overcome intrinsic GNN shortcomings. One of the simplest ways to integrate expert knowledge is to combine the expert-crafted descriptors with GNN-learned representation through concatenation.^17,18^ However, while commonly used, a thorough evaluation of this concatenation strategy is lacking. Furthermore, contextualizing this approach against existing virtual screening methods—both traditional and ML-based—is essential to understand its practical utility. Our study does not aim to claim superiority over all other approaches but instead investigates how GNN-based models, when integrated with domain knowledge, can become competitive with state-of-the-art alternatives.

This work contributes to the field by comprehensively evaluating this commonly used strategy in a virtual screening setting using nine well-curated HTS data sets. We find that although this strategy is often effective, it is not always the case. Additionally, we discover that the combined GNNs show convergence of performance metrics, suggesting the potential interchangeability of sophisticated GNN architectures with simpler counterparts under this integrative strategy. Moreover, surprisingly we found that descriptors are fairly robust under the scaffold split scenario, which is often a more realistic setting in a drug discovery campaign. These findings prompt the need to examine the current integration strategies to understand their limitations, find better ways to integrate domain expert knowledge and provide a path for more advanced ligand-based virtual screening.

## 2 Results

### 2.1 Concatenate Descriptors with GNNs

As shown in Figure 1, the concatenation strategy^17,18^ examined in this work proposes to train a neural network to predict activity by combining a GNN-derived molecular representation with the expert-crafted descriptors.^19^ Specifically, for a representation *h* from the GNN, it is concatenated with the descriptor *h*_*dp*_.

h=GNN(m)

$$\mathrm{p̂}=f\left([h\left|\right|h_{\mathrm{dp}}]\right)$$

where *m* is the input molecular graph and *h*_*dp*_ is a descriptor. *f*(·) is a classifier, usually a Multi-Layer-Perceptron (MLP). *p̂* is the predicted activity.

**Figure 1:**
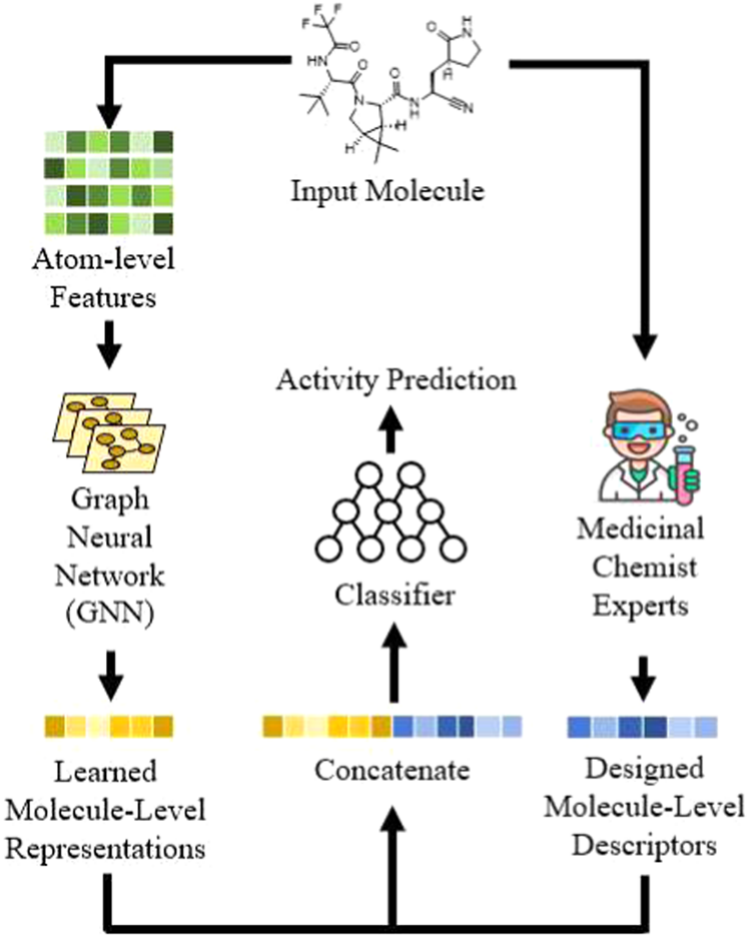
Overview of the investigated method. The learned molecular representation of GNN is concatenated with expert-crafted descriptors to enhance the predictive power.

The model is trained by optimizing the binary cross entropy loss *L*

$$L=-\frac{1}{n}\sum_{i=1}^{n}y_{i}\log\left(\mathrm{p̂}\right)+\left(1-y_{i}\right)\log\left(1-\mathrm{p̂}\right)$$

where *n* is the number of samples in a batch, and *y_i_* is the experimentally determined active/inactive status of the *i*-th molecule.

In this work, we used three GNN models in our experiments: GCN,^20^ SchNet^11^ and SphereNet.^13^ We used the BioChemical Library (BCL)^21^ to generate descriptors.

### 2.2 Effectiveness of the Concatenation Strategy Varies for GNNs with Random Split

In Figure 2 the boxplots of model performances evaluated using four different metrics are shown (experiments are detailed in Section 3). The *p*-value is calculated using paired *t*-test.^22^ For each data set and evaluation metric, we computed the mean performance difference between models with and without BCL descriptors. To assess the statistical significance of these differences, we applied paired *t-*tests and reported the corresponding 95% confidence intervals. To account for multiple comparisons across models and metrics, false discovery rate (FDR) adjustments were applied to all p-values. The effect sizes, 95% confidence intervals, and FDR-adjusted p-values are provided alongside the performance distributions in Figure 2. This statistical analysis provides a rigorous quantitative foundation to support the claims of performance improvement attributed to the descriptor integration strategy.

**Figure 2:**
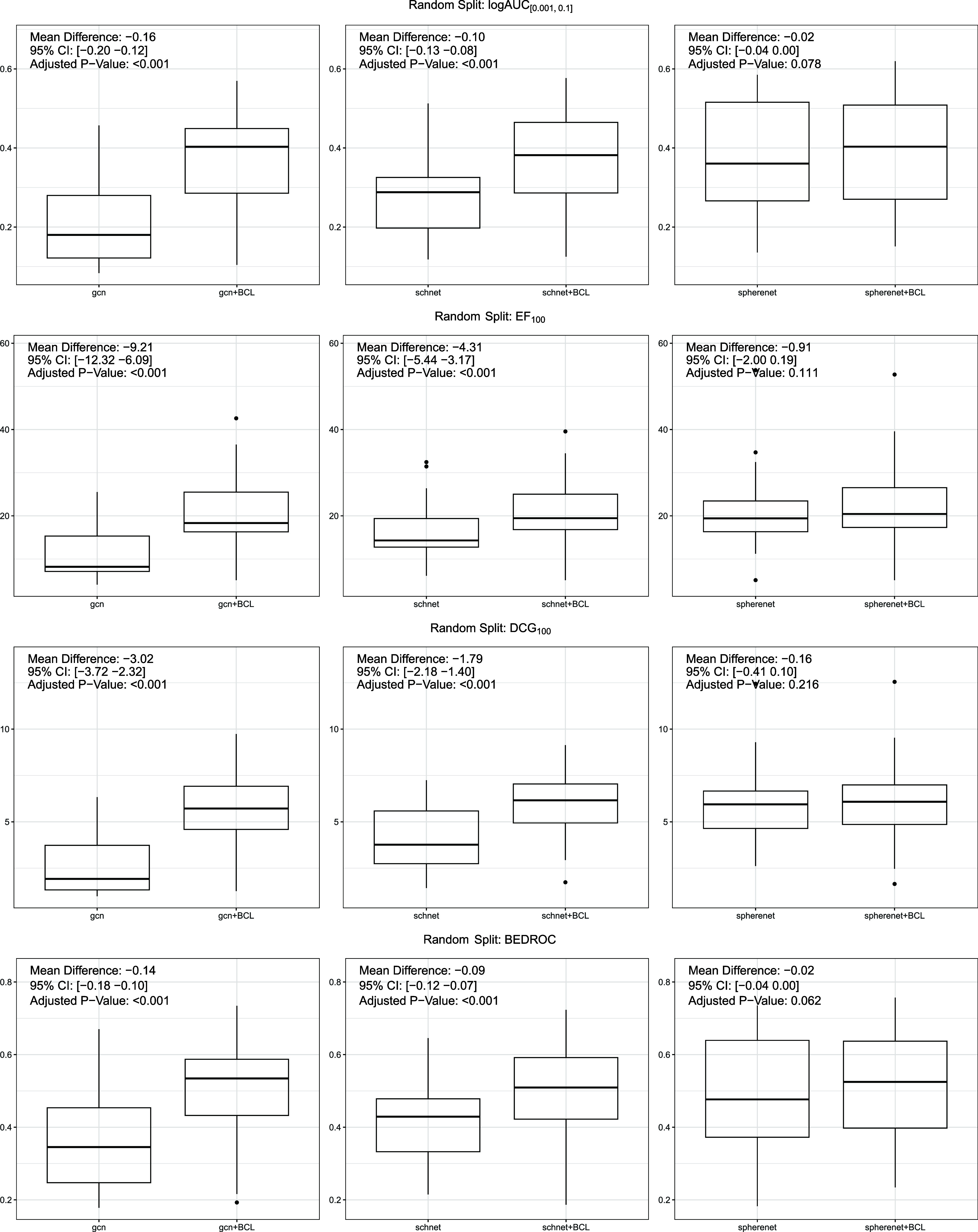
Random Split: Performance of three GNNs with their corresponding descriptor-integrated counterpart.

The significant improvements observed in both GCN and SchNet models across four evaluation metrics highlight the investigated strategy’s potential to facilitate the identification of bioactive compounds in drug discovery. Although the benefits were less pronounced for the SphereNet model (as a bigger *p*-value is observed), the overall results advocate for the integration strategy’s adoption as a valuable tool in computational chemistry.

There are three rationales for this approach. First, data available for training in drug discovery campaigns is usually limited due to the high cost of experimental assays. The expert-crafted descriptors supplement GNNs with prior knowledge, i.e., descriptors that worked well in virtual screening in the past, which reduces the need for GNNs to learn that knowledge from a large amount of data. Second, GNNs typically have difficulty learning molecular-level features due to their limited receptive field or learning nonadditive molecular-level features such as total polar surface area. On the other hand, molecular-level descriptors provide global features directly. Third, GNN intrinsically suffers from problems such as oversmoothing^14^ and oversquashing^15^ that introduce information loss in obtaining the global learned embedding from the atomic features. Meanwhile, the descriptors extract the molecular features directly and circumvent information loss, complementing GNN-learned embeddings.

### 2.3 All Descriptor-Integrated GNNs Converge to Similar Performance with Random Split

The analysis undertaken in this study revealed significant insight regarding the investigated strategy’s performance. Initially, the GNNs—each with its intrinsic computational complexities and capabilities—demonstrated disparate levels of efficacy. However, upon the integration of descriptors, a notable convergence in their performance metrics was observed, spanning all four evaluated metrics. As shown in Figure 2, SphereNet and SchNet, are more advanced GNNs compared with GCN. Yet, when these advanced GNNs were coupled with descriptors, the resultant performance was not just enhanced but aligned closely with that of their simpler counterparts GCN.

This intriguing outcome underscores the potency of the integration strategy in equalizing the performance landscape among GNN architectures. By integrating expert-crafted descriptors through the integration approach, even less complex GNN models could elevate their predictive accuracies to levels akin to those of more complex GNNs. Essentially, the integration strategy acts as a performance catalyst, diminishing the gaps between GNN models of varying complexities and facilitating a more uniform field of competition. Such findings highlight the potential of combining deep learning techniques with established domain knowledge, suggesting a reevaluation of the necessity for complex GNNs in scenarios where their simpler counterparts can achieve comparable outcomes through integration with descriptors.

### 2.4 Expert-Crafted Descriptor Still Outperforms Most GNNs Using Scaffold Split

Besides random split, we also conducted experiments on scaffold split. This is a realistic scenario because medicinal chemists often need to determine the activity of structures substantially different from those in the known training set. They seek these structural differences for various reasons, such as avoiding patented structures, finding simpler synthetic routes, improving compound properties etc.^23^

As expected, the overall performance under the scaffold split decreased compared with that under the random split. This decrease is due to the greater difficulty in predicting the performance of structures significantly different from the training set, as the data distribution differs between training and testing. However, as shown in Figure 3, the results from the scaffold split evaluation solidify the potential of the integration strategy in enhancing the performance of various GNN architectures for ligand-based virtual screening. The combined GNN-derived molecular representations with descriptors, improve the identification and prioritization of active compounds (Although outliers exist, which is consistent with our results for random split that the effectiveness of this strategy varies).

**Figure 3:**
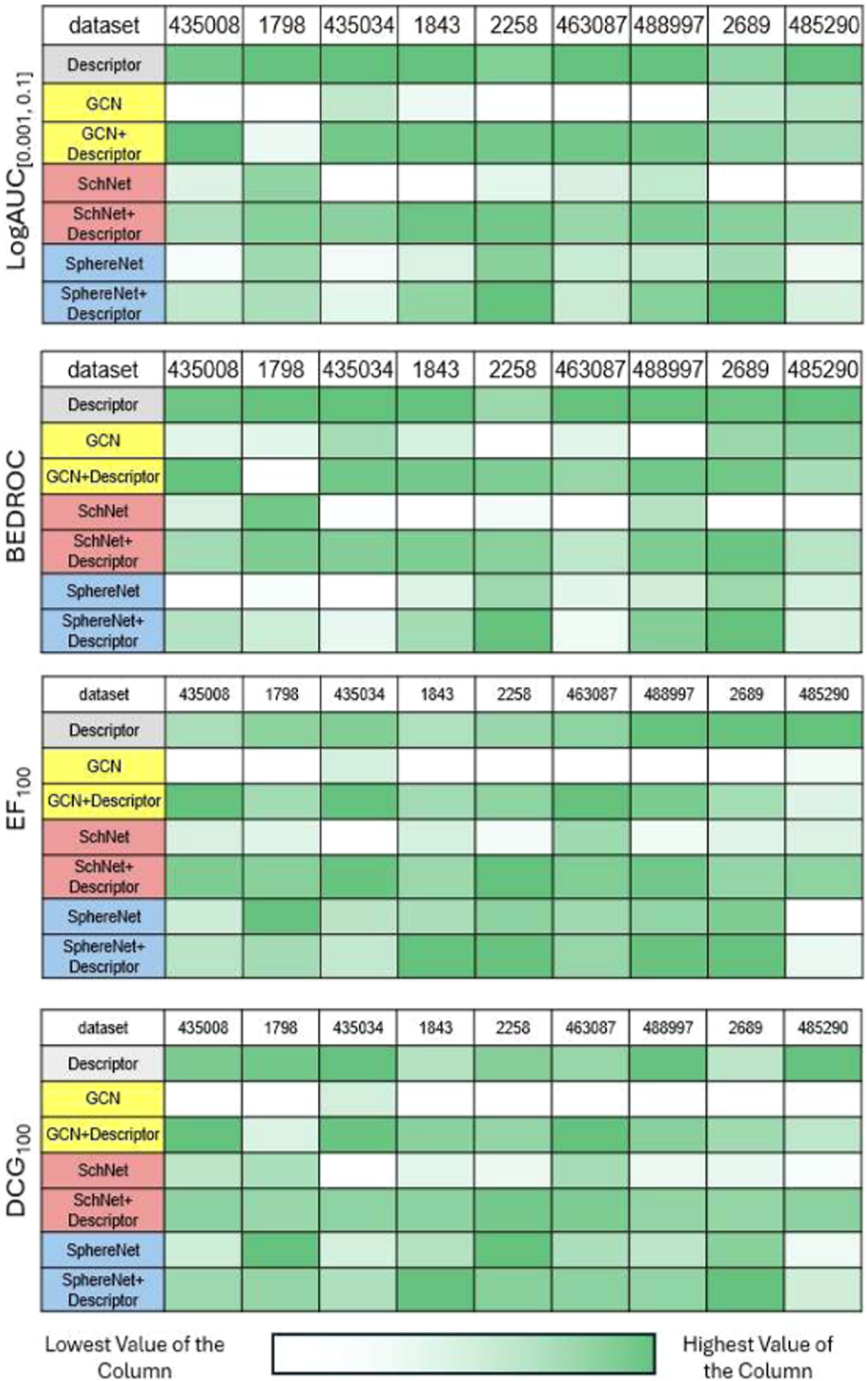
Scaffold Split: Performance of different models. The concatenation strategy still enhances the GNNs for most cases. Notably, descriptors perform better than many models across different metrics, especially salient in logAUC_[0.001,0.1]_ and BEDROC.

Most interestingly, we found that the descriptors alone outperform many GNNs. In some cases, it even outperforms the integrated-version GNNs. We hypothesize that this could result from the fact that deep learning-based methods are more easily overfit to the training data and therefore will perform worse than the expert-crafted ones when the data distribution is shifted. This finding prompts us to reconsider whether data-driven methods alone, despite their growing popularity, are the best approach for real-world drug discovery campaigns. Moreover, this also shows that even when coupled with descriptors, the performance of the integrated model may decrease and not always offer benefits. Finally, this finding emphasizes the need for developing better frameworks that integrate domain knowledge for improved predicted power under scaffold split scenarios.

The observation that expert-crafted descriptors sometimes outperform GNN-based models, particularly under scaffold split, may partially reflect overfitting in the learned representations. Given the limited number of active compounds and the distributional shift introduced by scaffold-based splitting, deep learning models—especially those with high capacity—are more prone to memorizing patterns specific to the training scaffolds. To reduce this effect, we applied standard regularization strategies such as dropout and avoided early stopping, instead selecting the model from the final training epoch as recommended in prior literature.^19^

To better contextualize our findings, we note that expert-crafted descriptor models, as evaluated in our study, already serve as strong baselines. This aligns with prior studies showing that traditional descriptor-based QSAR methods often outperform deep learning models in low-data or scaffold-split scenarios. Our results confirm this trend, particularly under scaffold split, where descriptors alone often outperform not only standalone GNNs but also the integrated versions. These findings suggest that while GNNs remain promising for virtual screening, especially when integrated with domain knowledge, traditional approaches are still competitive and in some cases preferable—highlighting the importance of hybrid strategies in current practice.

## 3 Methods

### 3.1 Data Sets

We validate the effectiveness of the proposed strategy via nine well-curated high-throughput screening (HTS) data sets. To avoid issues with experimental artifacts and high false positive rates,^24^ for the validation of our strategy, we chose data sets carefully curated^25^ from high throughput screens in the PubChem database.^26^ Only data sets with robust secondary validation of compounds were considered. Data set details are shown in Table 1.

**Table 1:** Data Set Statistics

protein target class	PubChem AID	protein target	total molecules	active molecules
GPCR	435008	Orexin1 Receptor	218,156	233
	1798	M1Muscarinic Receptor Agonists	61,832	187
	435034	M1Muscarinic Receptor Antagonists	61,755	362
ion channel	1843	Potassium Ion Channel Kir2.1	301,490	172
	2258	KCNQ2 Potassium Channel	302,402	213
	463087	Cav3 T-type Calcium Channels	100,874	703
transporter	488997	Choline Transporter	302,303	252
kinase	2689	Serine/Threonine Kinase 33	319,789	172
enzyme	485290	Tyrosyl-DNA Phosphodiesterase	341,304	281

SMILES from the data sets were converted to SDF files using Open Babel.^27^ Standardized 3D coordinates are generated using Corina.^28^ Molecules are further filtered with atom type validity and duplicates with the BioChemical Library (BCL).^21^

Random split is used for the experiments, and each data set is split into 80% for training and 20% for testing. Because preliminary results and previous literature^19^ have shown that dropout can help avoid overfitting and the number of known active compounds is limited, we take the model from the last training epoch instead of the one from early stopping determined by validation performance. Multiple splits are used to prove the robustness of the proposed strategy.

### 3.2 Evaluation Metric

#### 3.2.1 Logarithmic Receiver-Operating-Characteristic Area Under the Curve with the False Positive Rate in the range [0.001, 0.1] (logAUC_[0.001,0.1]_)

Ranged logAUC^29^ is used because only a small percentage of molecules predicted with high activity can be selected for experimental tests in consideration of cost in a real-world drug discovery campaign.^24^ This high decision cutoff corresponds to the left side of the receiver-operating-characteristic (ROC) curve, i.e., those false positive rates (FPRs) with small values. Also, because the threshold cannot be predetermined, the area under the curve is used to consolidate all possible thresholds within a certain small FPR range. Finally, the logarithm is used to bias toward smaller FPRs. Following prior work,^19^ we choose to use logAUC_[0.001,0.1]_. A perfect classifier achieves a logAUC_[0.001,0.1]_ of 1, while a random classifier reaches a logAUC_[0.001,0.1]_ of around 0.0215, as shown below

$$\frac{\int_{0.001}^{0.1}xd\log_{10}x}{\int_{0.001}^{0.1}1d\log_{10}x}=\frac{\int_{-3}^{-1}10^{u}du}{\int_{-3}^{-1}1du}\approx 0.0215$$

#### 3.2.2 Boltzmann-Enhanced Discrimination of Receiver Operating Characteristic (BEDROC)

BEDROC^30^ is a metric that evaluates the early recognition ability of a given model. It prioritizes the identification of active compounds early in the ranked list. BEDROC ranges from 0 to 1, where a score closer to 1 indicates better performance in recognizing active compounds early in the list.

#### 3.2.3 Enrichment Factor with Cutoff 100 (EF_100_)

Enrichment factor^31^ is a commonly used metric in virtual screening. It measures how well a screening method can increase the proportion of active compounds in a selection set, compared to a random selection set. Here we select the top 100 compounds as the selection set. And the EF_100_ can be defined as follows.

$${\mathrm{EF}}_{100}=\frac{n_{100}/N_{100}}{n/N}$$

where *n*_100_ is the number of true active compounds in the ranked top 100 predicted compounds given by the model, *N*_100_ is the number of compounds in the top 100 predicted compounds (i.e., 100), *n* is the number of active compounds in entire data set, *N* is the number of compounds in the entire data set. It is essentially a measure of the method’s ability to “enrich” the set of compounds for further testing.

A random selection set receives an EF_100_ of 1. If no true active compounds are in the top 100 compounds, the EF_100_ becomes 0.

#### 3.2.4 Discounted Cumulative Gain with Cutoff 100 (DCG_100_)

DCG^32^ is a measure of ranking quality often used in web search. In a web search, it is obvious that a method is better when it positions highly relevant documents at the top of the search results. Virtual screening has a similar evaluation logic where we desire the active molecules to appear at the top of the selection set.

To calculate DCG, a simpler version metric named cumulative gain (CG)^32^ is introduced below. CG is the sum of the relevance value of a compound in the selection set. In our case, a true active compound receives a relevance value of 1, while a true inactive compound receives a relevance value of 0. So, the CG with cutoff 100 (CG_100_) equals the number of true active compounds in the top 100 compounds, i.e.,

$${\mathrm{CG}}_{100}=\sum_{i=1}^{100}y_{i}$$

It can be observed that CG_100_ is unaffected by changes in the ordering of compounds. DCG hence aims to penalize a true active molecule appearing lower in the selection set by logarithmically reducing the relevance value proportional to the predicted rank of the compound, i.e.

$${\mathrm{DCG}}_{100}=\sum_{i=1}^{100}y_{i}/\log_{2}\left(i+1\right)$$

### 3.3 Baseline Models

We used three GNN models in our experiments: GCN,^20^ SchNet^11^ and SphereNet.^13^ The node and edges features can be found in the supplement. We used the BCL^21^ to generate traditional QSAR descriptors. Following previous examples,^19,33^ we use the optimal descriptors where 391-element molecular-level features are generated. We provide a brief introduction to each of the models and the BCL below.

GCN extends the concept of convolution from regular, grid-like data (such as images) to graphs, which have arbitrary structures. GCNs work by aggregating information from a node’s neighbors (potentially the node itself) to learn a representation of each node that captures both its features and local topology.

SchNet is a GNN designed for processing 3D molecules. The core design is continuous filters that are capable of handling unevenly spaced data, particularly, atoms. It also contains blocks that model interactions between atoms in a molecule.

SphereNet incorporates unique spherical message passing (SMP) for processing 3D molecules. It is encoded in a spherical coordinate system consisting of distance, angle and torsion. The SMP then uses the spherical coordinate system for the message passing process.

BCL is an application-based, open-source software package that integrates traditional small molecule cheminformatics tools with machine learning-based quantitative structure–activity/property relationship (QSAR/QSPR) modeling. It is designed to facilitate various cheminformatics tasks such as computing chemical properties, estimating druglikeness, etc. It serves as a valuable resource for researchers in the computer-aided drug discovery field by providing a modular toolkit that supports the integration of cheminformatics and machine learning tools into their research workflows.

## 4 Future Work

In future work, we plan to expand our investigation by incorporating a broader array of GNN architectures and descriptor sets. This expansion will allow us to evaluate the generalizability and scalability of our integrative approach across a wider spectrum of computational models and chemical descriptor libraries.

We aim to explore advanced GNN models that may offer distinct advantages in capturing molecular features and interactions, potentially leading to improved predictive performance in virtual screening tasks. By comparing a diverse range of GNN architectures, we can better understand the nuances of how different models interact with various descriptor sets, and identify optimal combinations that maximize screening efficacy and accuracy.

Another promising direction for future research is to investigate how different types of input features affect model performance. While our current study uses a fixed descriptor set that includes a mix of scalar, two-dimensional (2D), and selected 3D features, we did not systematically explore how each feature class contributes to performance. A more granular ablation of descriptor subsets—e.g., isolating scalar physicochemical properties, 2D topological descriptors, or 3D geometric features—could reveal how each dimension complements GNN-based representations. Similarly, for GNN input features, evaluating the impact of excluding or modifying certain atomic or bond features could provide deeper insights into model behavior and generalizability. We consider this a valuable direction for future work to further refine feature design and integration strategies.

Ultimately, our goal is to develop a comprehensive framework that can adapt to the evolving landscape of drug discovery, accommodating new advances in machine learning and cheminformatics.

## 5 Conclusions

Our study has rigorously evaluated the impact of integrating expert-crafted descriptors with GNNs and demonstrated that this integrative approach can significantly enhance the predictive power of virtual screening processes. Notably, the use of descriptors in conjunction with GNN architectures like GCN and SchNet has led to substantial improvements in identifying bioactive compounds. To assess the robustness and generalizability of our approach, we conducted experiments across nine well-curated high-throughput screening (HTS) data sets, encompassing a diverse set of protein targets from five major classes: GPCRs, ion channels, transporters, kinase, and enzymes. These data sets were selected to reflect realistic virtual screening (VS) scenarios, particularly through scaffold-split evaluations that simulate generalization to novel chemotypes.

In addition, the convergence in performance metrics across different GNN models, when supplemented with descriptors, suggests the potential for simpler GNN architectures to achieve results comparable to their more complex counterparts within this integrative framework. This finding underscores the viability of leveraging traditional knowledge and computational simplicity to advance the state-of-the-art in virtual screening.

Furthermore, our experiments with scaffold split scenarios revealed the robustness of descriptors, often outperforming combined GNN-descriptor models. This highlights the enduring value of expert knowledge in the face of evolving computational techniques and stresses the necessity for future models to effectively integrate this knowledge to enhance predictive power in realistic drug discovery settings.

In conclusion, our study serves as a compelling demonstration of how the synergistic integration of GNNs and expert-crafted descriptors can significantly advance the field of ligand-based virtual screening. As we move forward, it is imperative that we continue to explore and refine these integrative strategies, with the aim of developing more sophisticated and effective tools for drug discovery. The journey toward optimizing virtual screening methodologies is far from complete, but our work provides a significant step forward, offering a blueprint for future research in this dynamic and evolving field.

## Supplementary Material



## Data Availability

The data used in this work is from ref 25 and can be freely downloaded at https://figshare.com/articles/dataset/Well-curated_QSAR_datasets_for_diverse_protein_targets/20539893. Software and instructions are detailed in the supplementary protocol capture.

## Notes

Details of descriptor features; node and edge features for the GNNs (PDF)
