# Supplementary material for: Advancements in Ligand-Based Virtual Screening through the Synergistic Integration of Graph Neural Networks and Expert-Crafted Descriptors
Source: J Chem Inf Model. 2025 May 14;65(10):4898–905. doi: 10.1021/acs.jcim.5c00822 (PMC12117557; doi:10.1021/acs.jcim.5c00822)
Supplement: Supplementary file 1 [file ci5c00822_si_001.pdf]

Advancements in Ligand-Based Virtual Screening through the Synergistic Integration of Graph Neural Networks and Expert-Crafted Descriptors

Yunchao (Lance) Liu

Department of Computer Science, Vanderbilt University, 2201 West End Ave
Nashville, Tennessee 37235, USA
yunchao.liu@vanderbilt.edu

Rocco Moretti

Department of Chemistry, Center for Structural Biology, Vanderbilt University, 2201 West End Ave
Nashville, Tennessee 37235, USA
rocco.moretti@vanderbilt.edu

Yu Wang

School of Computer and Data Sciences, University of Oregon, 1585 East 13th Avenue,
Eugene, Oregon 97403, USA
yuwang@uoregon.edu

Ha Dong

Department of Neural Science, Amherst College, 220 South Pleasant Street
Amherst, Massachusetts 01002, USA
hdong26@amherst.edu

Bailu Yan

Department of Biostatistics, Vanderbilt University, 2201 West End Ave
Nashville, Tennessee 37235, USA
bailu.yan@vanderbilt.edu

Bobby Bodenheimer

Department of Computer Science, Electrical Engineering and Computer Engineering, Vanderbilt University, 2201 West End Ave
Nashville, Tennessee 37235, USA
bobby.bodenheimer@vanderbilt.edu

Tyler Derr1

Department of Computer Science, Data Science Institute, Data Science Institute, Vanderbilt University,
2201 West End Ave
Nashville, Tennessee 37235, USA
tyler.derr@vanderbilt.edu

Jens Meiler1

Department of Chemistry, Center for Structural Biology, Vanderbilt University, 2201 West End Ave
Nashville, Tennessee 37235, USA
Institute of Drug Discovery, Leipzig University Medical School, Härtelstraße 16-18,
Leipzig, 04103, Germany
Center for Scalable Data Analytics and Artificial Intelligence (ScaDS.AI), Humboldtstraße 25,
Leipzig, 04105, Germany
jens.meiler@vanderbilt.edu

---

1 Corresponding Authors

Supporting Information

Descriptor Features

The descriptor sets used in this study are adapted from [1]. The descriptor used in the domain baseline is generated with BCL [2]. There are three types of features in the generated descriptor: scalar, signed 2D autocorrelations (2DA_Sign) [3], and signed 3D autocorrelations (3DA_Sign} [3].

The original unsigned version of 2D_Sign is proposed in [4] (denoted as 2DA). The original unsigned version of 3D_Sign is proposed in [5] (denoted as 3DA). 2DA defines bond distance as the distance measure, while the 3DA defines the 3D Euclidean distance as the distance measure. The 2DA and 3DA calculate the autocorrelation value for a distance bin between r_a and r_b based on the following formula:

$$Autocorrelation(r\_a, r\_b) = \sum_{i}^{n}\sum_{j}^{n} \mathbb{1}(r_{ij})P_iP_j$$

where i and j are two nodes (atoms), n is the total number of nodes, P is an atomic property, $\mathbb{1}(\cdot)$ is an indicator function, which evaluates to 1 if the distance r_{ij} satisfies $r_a < r_{ij} < r_b$, otherwise evaluates to 0.

2DA_Sign and 3DA_Sign extend the original 2DA and 3DA by having 3 values for each distance bin, corresponding to the positive-positive, positive-negative, and negative-negative pairs of atomic properties, to circumvent the situation where a negative and a positive cancel out during the summation. The atomic properties used in 2DA_Sign and 3DA_Sign are listed in **Table S1**.

There are 23 scalars, each taking one dimension.

The 2DA_Sign evaluates up to 11 bonds (exclusive). It generates 32 one-dimensional values across 11 distance bins (initially resulting in 33 dimensions, but the first bin, which represents 0 bonds away, corresponds to the square of an atom's property and lacks positive-negative pairs, so it is excluded). With 4 atomic properties considered, the 2DA_Sign consists of 32 values per property, resulting in 128 dimensions.

The 3DA_Sign evaluates up to 6 Å (exclusive). There are 24 distance bins for a step size of 0.25 Å. However, the distance bin [0, 0.25) reduces to 2D_Sign, therefore excluded. [0.25, 0.5), [0.5, 0.75], [0.75, 1) are generally evaluated to 0, therefore are excluded as well. This results in a total number of 20 distance bins. Each bin has 3 values, so there are 60 one-dimensional values for each atomic property. With 4 atomic properties, there are 60*4=240 dimensions for 3DA_Sign.

The final descriptor is of 391 dimensions (23+128+240 = 391).

Table S1. Features used in the descriptor set. Originally used in [1].

Feature Type	Feature Explanation
**Scalar**	Molecular Weight
	Number of Hydrogen Bond Donors
	Number of Hydrogen Bond Acceptors
	LogP – Octanol/Water Coefficient [6]
	Total Charge of the Molecule
	Number of Rotatable Bonds
	Number of Aromatic Rings

	Number of Rings
	Topological Polar Surface Area (TPSA) [7]
	Girth (Widest Diameter of Molecule in Å)
	Bond Grith (Maximum Number of Bonds Between Two Atoms)
	Number of Atoms in the Largest Ring
	Number of Atoms in the Smallest Ring
	Number of Bridge Atoms in Fused Aromatic Ring
	Number of Bridge Atoms in Fused Ring
	Max of Atom V Charge [8]
	Min of Atom V Charge
	Standard Deviation of Atom V Charge
	Sum of Absolute Value of V Charge
	Max of Atom σ Charge [9]
	Min of Atom σ Charge
	Standard Deviation of Atom σ Charge
	Sum of Absolute Value of V Charge
Signed 2D Autocorrelation	Atom σ Charge
	Atom V Charge
	Hydrogen Or Heavy Atom
	Whether In Aromatic Ring
Signed 3D Autocorrelation	Atom σ Charge
	Atom V Charge
	Hydrogen Or Heavy Atom
	Whether In Aromatic Ring

Node and Edge Features

Table S2 Edge features used in GNNs, adapted from [10]

Index	Description
0	Is aromatic
1	Is conjugate
2	Is in a ring
3-6	One-hot encoding of bond type: 1, 1.5, 2, 3

References

1. Mendenhall, J. and J. Meiler, *Improving quantitative structure–activity relationship models using Artificial Neural Networks trained with dropout.* Journal of computer-aided molecular design, 2016. **30**(2): p. 177-189.
2. Benjamin P. Brown, O.V., Alexander R. Geanes, Sandeepkumar Kothiwale, Mariusz 4 Butkiewicz, Edward W. Lowe, Jr., Ralf Mueller, Richard Pape, Jeffrey Mendenhall, and Jens Meiler, *Introduction to the BioChemical Library (BCL): An application-based open-source toolkit for integrated cheminformatics and machine learning in computer-aided drug discovery.* 2022.
3. Sliwoski, G., J. Mendenhall, and J. Meiler, *Autocorrelation descriptor improvements for QSAR: 2DA_Sign and 3DA_Sign.* Journal of computer-aided molecular design, 2016. **30**(3): p. 209-217.
4. Moreau, G., *The autocorrelation of a topological structure: A new molecular descriptor.* 1980.
5. Broto, P., G. Moreau, and C. Vandycke, *Molecular structures: perception, autocorrelation descriptor and SAR studies. Perception of molecules: topological structure and 3-dimensional structure.* European journal of medicinal chemistry, 1984. **19**(1): p. 61-65.
6. Xing, L. and R.C. Glen, *Novel methods for the prediction of logP, p K a, and logD.* Journal of chemical information and computer sciences, 2002. **42**(4): p. 796-805.
7. Ertl, P., B. Rohde, and P. Selzer, *Fast calculation of molecular polar surface area as a sum of fragment-based contributions and its application to the prediction of drug transport properties.* Journal of medicinal chemistry, 2000. **43**(20): p. 3714-3717.
8. Gilson, M.K., H.S. Gilson, and M.J. Potter, *Fast assignment of accurate partial atomic charges: an electronegativity equalization method that accounts for alternate resonance forms.* Journal of chemical information and computer sciences, 2003. **43**(6): p. 1982-1997.
9. Gasteiger, J. and M. Marsili, *Iterative partial equalization of orbital electronegativity—a rapid access to atomic charges.* Tetrahedron, 1980. **36**(22): p. 3219-3228.
10. Coley, C.W., R. Barzilay, W.H. Green, T.S. Jaakkola, and K.F. Jensen, *Convolutional Embedding of Attributed Molecular Graphs for Physical Property Prediction.* Journal of Chemical Information and Modeling, 2017. **57**(8): p. 1757-1772.
